# Strategies to Reduce Salt Content and Its Effect on Food Characteristics and Acceptance: A Review

**DOI:** 10.3390/foods11193120

**Published:** 2022-10-07

**Authors:** Siti Nurmilah, Yana Cahyana, Gemilang Lara Utama, Abderrahmane Aït-Kaddour

**Affiliations:** 1 Faculty of Agro-Industrial Technology, Universitas Padjadjaran, Jalan Raya Bandung-Sumedang Kilometer 21, Jatinangor 45363, Indonesia; 2 Center for Environment and Sustainability Science, Universitas Padjadjaran, Jalan Sekeloa Selatan I No. 1, Bandung 40134, Indonesia; 3 VetAgro Sup, INRAE (National institute for Agriculture, Food, and Environment), Université Clermont-Auvergne, 63370 Lempdes, France

**Keywords:** salt reduction, strategy, hypertension, salty food, low salt

## Abstract

Sodium is a necessary nutrient for regulating extracellular fluid and transferring molecules around cell membranes with essential functions. However, the prevalence of some diseases is related to unnecessary sodium intake. As a result, a particular problem for the food industry remains a matter of sodium content in foods. It is considered that customer acceptance is associated with salt perception dynamics related to the evolution of food production. It is a significant challenge and technique to minimize the salt content of various foods and provide replacement products with substantial reductions in salt levels. This review summarizes salt reduction strategies related to health problems based on traditional review methodology, with practical and methodological screening performed to determine the appropriate reference sources. Various technological (salt replacement, food reformulation, size and structural modifications, alternative processing, and crossmodal odor interaction) and behavioral strategies (memory process, gradual salt reduction, and swap) are identified in this work, including a deeper understanding of the principles for reducing sodium content in foods and their effect on food characteristics and potential opportunities for the food industry. Thereby, the food industry needs to find the proper combination of each strategy’s advantages and disadvantages to reduce salt consumption while maintaining product quality.

## 1. Introduction

Health authorities suggest that dietary salt should be gradually reduced because excessive sodium intake causes many diseases. High salt intake is correlated with cerebrovascular, heart disease, ventricular hypertrophy, kidney injury, and other damage to the target organs [1,2,3,4]. Salt contains 40% sodium and 60% chloride. Table salt provides approximately 90% of the sodium in the diet [2]. About 75% of salt consumption (NaCl) comes from processed foods, not only for sensory but also for microbiological problems avoidance [4,5,6,7].

The World Health Organization (WHO) proposes to lower NaCl intake in targeted foods by 35% by 2025, such as bread, dairy products, soups, cheeses, meats, fish, and other foods. In most cases, sodium consumption is well above the recommended intake level. Therefore, reducing the amount of sodium intake in foods remains an essential concern for the food processing industry. However, in most countries, dietary salt consumption is well above the threshold level of <5 g/day, and salt restrictions on population consumption have been rated as one of the least expensive interventions to minimize cardiovascular disease [8]. Several national salt reduction initiatives, including interventions in schools, workplaces, fast food chains or restaurants, hospitals, and government offices and other arrangements, food reformulation, front-of-pack labeling, consumer education, and salt taxation, demonstrate the seriousness of efforts to reduce daily salt intake [9,10,11,12,13,14,15,16].

Efforts to reduce salt consumption gradually and sustainably through salt reduction programs have been successful in several countries, including the United Kingdom (UK), Finland, Poland, and Japan. The most successful nutritional program is the UK salt reduction model. The method involves gradually changing the salt reduction of more than 80 food categories produced by the industry over a set period [17]. Over four years, this strategy successfully lowered salt by at least 16% [18]. This accomplishment is measured by the fact that individuals do not notice a difference in the taste of food that its salt content gradually lowered. In Finland and Poland, salt reduction reduced the prevalence of stroke by 10.1% and 23.1%, respectively [19]. Furthermore, Japan managed to decrease salt intake from 14.5 g to 9.5 g from 1973 to 2017 [20]. These encouraging results have to be applied in other countries.

Furthermore, salt reduction can have an impact on food characteristics as well as on customer acceptance. Consumer taste is associated with the complexity of salt perception. The challenge of salt reduction is that NaCl plays multiple roles in food products. Salt is a vital tool for controlling water activity (A_w_) in food preservation, delivering nutrients, and serving as a source of electrolytes [21,22,23,24]. An inappropriate salt reduction will impact food characteristics and consumer perceptions. As a result, a decrease in salt content will reduce global food acceptance, leading to a decline in interest and a negative economic effect—changes in food preparation connected with the intake of salty foods [25]. Thus, the primary challenge is to lower the salt concentration while maintaining acceptability [26].

Strategies that are further advanced from the technological aspects of reformulation, replacement, substitution, particle size modification, advanced processing technology, memory process, or behavioral aspect, such as gradual salt reduction, swap, and further traces of consumer perception of salt in food, are growing. This review aims to provide an overview of salt reduction strategies and their effect on food characteristics based on published research. Various databases, including Science Direct, Google Scholar, and Web of Science, were used to conduct this review. The review was limited to published reports, experiments, book chapters, and review papers. It goes through the ideas for lowering the salt levels in foods, their relevance to health, and their impact on food characteristics.

## 2. Salt Content and Contribution to Various Food Products

Salt or sodium chloride (NaCl) is a specific food ingredient commonly used in food service and common food processing. In some countries, there is usually a label on packaged products that indicates the salt content used in the product. The salt content category in products is classified into several sodium groups per serving. There are sodium-free (<5 mg), very low (<35 mg), low (<140 mg), reduced sodium (<25%), light sodium (<50%), and no salt added during processing [27]. However, a front-of-pack label promoting salt reduction may have a negative impact on salt use and taste perception [28]. It is important to note that different countries have different regulations for salt labeling [29]. According to various sociodemographic variables, information on salt content varies among countries. In some countries, the terms sodium and salt content are used on food labeling. For instance, Malaysia uses salt levels listed on the food label [30]. In comparison, the Percentage of Nutrient Reference Value (%NRV) is used in China [31]. However, a study in 12 countries showed that consumers were confused about dietary guidelines and the connection between salt and sodium [32]. This result remains a challenge in food labeling concerning efforts to reduce salt intake. In order to discourage consumers from consuming a high salt intake, reliable and effective food labeling is important for customers to guide them to choose healthier food [33].

Fresh foods usually have moderate amounts of sodium, while processed food products mainly contribute to the dietary sodium intake [34]. For example, eggs and milk contain 80 and 50 mg/100 g of sodium, while processed foods, such as pizza, bacon, bouillon cubes, soy sauce, and pretzels, may contain about 250–20,000 mg/100 g [27]. Food-dense and some solid freezes, such as pizza, cheese, and sausages, are the saltiest in the food segment [35]. Of the dominant contributors to sodium purchased by consumers, 23% were table salt, 18% cooked meat, 13% bread and bread products, 12% dairy products, and 11% sauces and spreads [36].

Salt is widely used to provide a salty taste, improve flavor, and act as a preservative. Salt is a major contributor to human food consumption due to its widespread use in foods. Salt inhibits certain enzymatic reactions in foods, which contributes to activating reactions that facilitate the characterization of color, texture, and taste properties [37,38]. Salt also adds flavor and bitter taste masks in foods, regulates yeast and the growth of fermented bacteria, and promotes proteins or other binding components in foods to achieve the desired texture [12,34]. In application, salt has technical properties, such as the arrangement of meat and pasta, the preservation of pickles, meat, margarine and milk spoilage, and changes in enzyme production in cheese [1,37]. In terms of preservatives against spoilage microorganisms and foodborne pathogens, NaCl is the sodium-containing molecule most effectively used in food and impacts food safety and quality in a microbiological view [4,37]. The salt content in the water phase of food affects the microorganisms. Preservation mechanisms also include redox potential and chemical preservatives. Salt’s osmotic impact is responsible for changing the metabolism of foodborne pathogens, spoilage microorganisms, and preserving various components of foods [21]. This result is related to the shelf life of products because the addition of sodium ions to the products causes water to flow through the semipermeable membrane of bacteria, resulting in water loss from cells and osmotic shock, which leads to bacterial cell death or serious injury, thereby resulting in a significant reduction in bacterial growth [39]. Another mechanism of salt related to extending the shelf life is the role of salt as an essential tool for controlling A_w_ in food, which prevents the growth of bacteria that can extend food shelf life [40].

## 3. Strategies to Reduce Salt Content in Various Food Products

To meet Na^+^ and Cl^−^ population intake objectives, systemic programs should encourage health- and technology-based awareness, experience, and skills relevant to salt intake reduction [2,14,41,42]. However, reduced salt or salt substitutes often produce poor sensory quality [39,43]. Due to the significant contribution of food to dietary salt intake, various technical strategies to develop low sodium foods without compromising food quality are being developed. Chemical stimulation to increase salt taste in the periphery, cognitive mechanisms to increase sensitivity or change salt tolerance, and product structures designed to maximize salt distribution to the tongue to increase the salty taste are the principal strategies that can be undertaken [21]. Therefore, providing alternative processes and techniques with significant salt content reductions is an important challenge in the food industry. In general, salt reduction strategies are related to technological and behavioral aspects.

### 3.1. Technological Strategy

#### 3.1.1. Salt Replacement Strategy

One of the salt replacement strategies can be replacing salt using cations that are beneficial for blood pressure. Potassium can reduce some of the adverse effects of a high sodium intake. Lowering blood pressure is most likely due to a substantial increase in potassium and a reduction in average sodium intake [44]. Charlton et al. [45] successfully reduced salt by 32% and partially replaced it with cations known to lower blood pressure, notably potassium (K), magnesium (Mg), and calcium (Ca), without compromising bread quality [45]. Salt substitutes can also increase flavor to minimize sodium levels by at least 25% and concurrently increase the content of calcium chloride (CaCl_2_), potassium chloride (KCl), or magnesium chloride (MgCl_2_) [45,46]. Meanwhile, NaCl partially replaced with other electrolytes allows for maintaining electrolyte levels necessary to optimize process efficiency and has potential health benefits rather than simply reducing NaCl. However, substituting KCl in the diet has serious and potentially fatal repercussions for people who need to limit their potassium intake. One third of Australian Chronic Kidney Disease patients exceed the safe limit for dietary potassium consumption when NaCl in bread is replaced by KCl (20–40%) [47]. As a result, improved food labeling is required to help customers avoid excessive consumption.

NaCl substitution using anions, glutamate, and adenosine is more effective than other anions in inhibiting bitterness related to sodium cation [48,49]. A low-sodium diet using umami seasoning (L-glutamate) was reported by Kawano et al. [50] in a single-blind crossover intervention study. Clinical schizophrenics were given a low-sodium diet with monomagnesium di-L-glutamate and had a 25.9% reduction in dietary sodium. In addition, no decrease in daily energy intake and no significant changes in body mass index, body weight, blood pressure, abdominal circumference, or nutrient intake were observed [50]. Some yeast extracts, which have a taste without contributing any additional odor, can also replace salt in food. According to Zheng et al. [51], the salty peptide fractionation of FA31 (Angel Yeast) could be determined through ultrafiltration, gel permeation chromatography, and preparative fluid chromatography (pre-HPLC) using a sequence of salty peptide components, including Asp-Asp, Glu-Asp, Asp-Asp-Asp, Ser-Pro-Glu, and Phe-Ile. According to the typical characteristics of the five peptide sequences, Asp-Asp and Glu-Asp have salty, umami, and sour tastes; Asp-Asp-Asp has a salty and an umami taste; Ser-Pro-Glu has a salty and sour taste, and Phe-Ile has a salty and bitter taste [51]. The incorporation of 5% yeast extract indicated that the formulation for promoting healthier salted salmon with good sensory acceptability and low sodium content could be used [52].

In addition, herbs, spices, and mixes also impart novel flavors and sensory sensations that may mask the absence of salt. Several plant-derived seasonings (e.g., garlic, herb blends, saffron, deadnettle family, and spicy spices) have shown good consumer acceptance when applied as salt substitutes [53]. Many different types of herbs can still be used as a seasoning with ethnic characteristics. Lovage, for example, is a popular flavoring ingredient used for salt substitutes [54]. This result proves that the development of a salty perception by flavor boosters and aromas can decrease salt intake [21]. The replacement urges for improved technological performance to be maintained.

#### 3.1.2. Food Reformulation Strategy

Food reformulation could play a significant role in rebalancing dietary consumption [55]. Some antagonistic and synergistic sodium reduction effects in complex food products have also been investigated. For example, the considerable influence of salt perception shows that salt, as opposed to fat, plays a significant role in the attraction of savory fatty foods [56]. However, acid flavors, such as citric, lactic, and tartaric acids, can enhance the perception of saltiness at low concentrations while having no impact or suppressing it at high concentrations; this result is related to pairwise interactions among salty, sour, and bitter elements accounting for a significant fraction (∼30–50%) of the potential binary taste interactions [57]. Although there is a significant correlation between perceived sweetness and suppression of salt perception in cream-based products, studies reveal that lactose or dry glucose syrup reduces salt perception through taste–taste interactions due to the interaction between sweetness and viscosity [22].

In emulsion-based foods, saltiness can increase with increasing fat and salt content concentrations in the aqueous phase. In this case, the use of unsaturated and essential fats appears to be beneficial. In addition to increasing the salty taste with reduced salt content, it can also provide consumers with health benefits. For example, by increasing the concentration of canola oil up to 40%, the salty intensity of NaCl and KCl will increase [58]. A water-in-oil (W/O) emulsion would appear less salty than an oil-in-water (O/W) emulsion [59]. By adjusting the mass fraction of the aqueous phase, the water-in-oil emulsion (W/O) saltiness perception can be modified [60]. According to the emulsion’s formula structure, more research is still needed to investigate the O/W saltiness and W/O emulsion. While internalized salt stabilized with gelatinized waxy rice starch can improve the salt reduction strategy of W/O/W emulsions food products, the aim is to release exposed salts due to amylase-induced instability during oral processing [61]. The encapsulated aqueous salt phase with octenyl succinic anhydride (OSA)-starch was studied in vitro, in vivo, and a sensory analysis revealed that it was feasible to reduce salt reduction by 23.7% without affecting the perception of saltiness [62]. Another study shows that double starch W/O/W quinoa starch granule pickering emulsion at 0.1 and 0.2 M salt encapsulation was able to maintain more than 90% stability after 21 days [63]. Thus, the reformulation strategy should focus on the balance of taste and product stability to produce a product that adheres to the suggested salt consumption while retaining the product’s features

#### 3.1.3. Modification of Size and Structural Strategies

**Figure 1:**
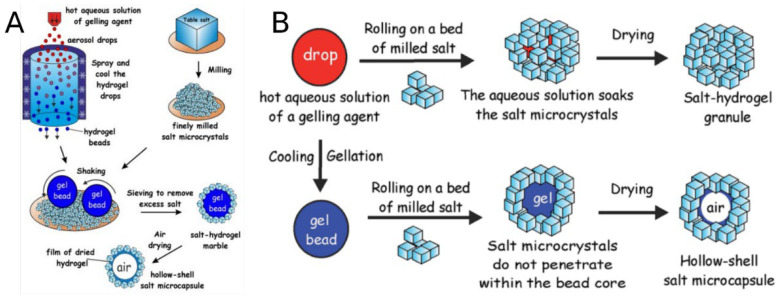
(**A**) Salt–hydrogel marbles and hollow-shell particle production; (**B**) Possible outcomes in the fabrication of salt marbles. Reproduced from Ref. [69] with permission from the Royal Society of Chemistry.

Spray drying, electromagnetic atomization drying, ultrasound, and other advanced technology were used to modify the size and structure of common salts. For instance, salt particles with a smaller size and lower bulk density can be produced by using a spray dryer. The substitution of NaCl with 30% KCl combined with spray dryer treatment with a lower feed flow rate resulted in salt particles with a higher salinity level [64]. Furthermore, hollow salt particles (~10 μm) produced by simple spray drying could be turned into vehicles for boosting flavor performance while lowering sodium intake and delivering hydrophobic bioactivity in food systems [65]. Moreover, the production of nanoscale salt crystal sizes of 520 nm using electromagnetic atomization drying (EAD) also increases saltiness and reduces sodium content by up to 65% in potato chip products [66]. Spray drying and atomization techniques are also used in a mixture of a salt dissolved in a solvent combined with a nonhygroscopic organic material (e.g., Gum Arab or maltodextrin) to produce a salt product size that is less than 100 μm [67]. The saltiness and higher dissolution of the maltodextrin/NaCl complex using spray drying were determined by atomization strength and inlet temperature [68]. One of the atomization techniques is the production of salt–hydrogel marbles from salt microcrystals and an aqueous gelling agent solution (Figure 1). Salt–hydrogel marbles form in cooling air columns by atomizing droplets of hydrogel solution, followed by hydrogel microbeads produced in beds of micrometer-sized salt particles [69].

Ultrasound techniques can also be used for atomization. Ultrasound treatment achieved a 0.75% decrease in salt size, resulting in a loss of about 30% of the sodium content [70]. Salt particles with a diameter of 20 μm have a greater dispersion in the food matrix, resulting in a saltier flavor [71]. Different types of physical salts are mixed with the size of salt crystals. The physical form of salt, the binding of surface area relative to volume, can produce a greater saltiness. Thereby, the product’s salt content can be reduced by 25–15% in different product applications [72].

**Figure 2:**
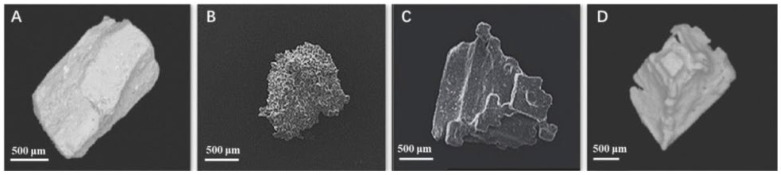
Various morphological forms of salt crystals: (**A**) cubic rock salts; (**B**) aggregate sea salts; (**C**) flaked sea salts; and (**D**) pyramidal sea salts. Reprinted with permission from Refs. [73,74,77]. Copyright 2022 Elsevier Licenses No. 5402170471962, 5402170729257, and 5402170140464.

Varying salt crystal morphologies have different porosities, solubilities, dissolution rates, and salt perceptions (Figure 2). There are some different salt crystal morphologies, including: (1) rock salts, which are regular cubes with a smooth surface, high density, and few cracks and pores; (2) aggregated sea salts, which are assembled entities of small agglomerated crystals with small crystals attached to large crystals; (3) flake salts, which have a larger surface area and low density; and (4) pyramidal sea salts, which have a hollow pyramid structure and a relatively rough surface [73,74]. The porous structure of the crumb is a strategy that can be used for salt reduction in bread because the coarse-porous bread shows a faster release of sodium than fine-porous bread [75]. The application of a hollowed microsphere of regular salt crystals on tuna and shrimp products is proven to maintain product quality [76].

**Figure 3:**
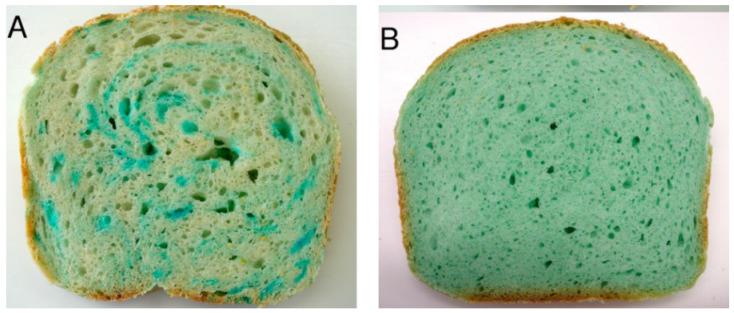
(**A**) Inhomogeneous sodium distribution of coarse-grained salt crystals (2−3.5 mm, 1.25%) and (**B**) homogeneous sodium distribution of salt crystals (<2 mm, 1.5%) Adapted with permission from [79]. Copyright 2013 American Chemical Society.

The heterogeneous distribution of salt in foods is a viable method for developing foods with decreased salt content while preserving the desired texture and taste [78]. This technique was reported by Li et al. [35] in that sensory evaluations showed an increase in salty semisolid food with an inhomogeneous salt distribution that decreased sodium levels by 30% with maintained flavor and texture properties. Inhomogeneous sodium distribution in bread using coarse-grained NaCl also greatly increased sodium release and salt taste, as shown in Figure 3 [79].

Moreover, encapsulated salt crystals cause the spatial distribution of salt in a solid product, such as bread, to be inhomogeneous, with local zones of high salt concentrations. Small encapsulates (1000 μm) provide salt concentration gradients that allow for a salt reduction of 50% and have no effect on saltiness intensity or customer acceptance [80]. Another study shows that encapsulation is an efficient way of maintaining high-salted spots. A total of 25% of encapsulated salts create a high-salted area and allow a salt reduction of 50% [81]. The encapsulating material also has a large influence on salt dissolving. Wax is regarded to be more effective than fat in inhibiting salt granule dissolution [77]. Furthermore, the encapsulation technology for nonvolatile oleoresin compounds may provide standardized taste and aroma products for salt reduction in food systems through different techniques [82]. Sensory contrast structures and a faster sodium release are a function of sodium’s kinetic release when chewing, suggesting that Arabic gum induces the swelling of the mucin layer to increase salinity and the acceleration of sodium diffusion with the Arabic coacervate protein/gum [35].

#### 3.1.4. Alternative Processing Strategy

##### High-Pressure Processing (HPP)

The advanced processing technology is a viable option for reducing salt in food. The high-pressure processing (HPP) technique is often used to tenderize fresh meat while forming a stable structure of processed meat [83]. HPP can boost protein solubilization, reducing cooking loss, and improve salt distribution to produce sodium-reduced meat [84,85,86]. Two stages of HPP at 300 and 600 MPa employed in ready-to-eat chicken breasts reduced salt content up to 50% with enhanced product quality and microbiological safety [84], while the pressure intensity of 200 MPa, in combination with heating, can be utilized to make the required gel product, for instance, treatment on meat dough can produce meat products with a low salt gel type [86]. At a pressure of 200 MPa, more free water is attracted by the protein or trapped in the gel structure than transferred to bound or immobilized water [85]. However, HPP used in processing pork with a low salt content (0.5–2.5%) at 150 MPa for 5 min shows that while there were adverse effects on color, texture, supination, and firmness, sensory levels of up to 2% were still acceptable [39]. In contrast, HPP at 600 MPa on ham and dried-cured pork increases salty levels in meat without adding salt concentration [87]. HPP was also successfully applied at 300 MPa for 3–5 min at 4–25 °C in meat products before cooking with a reduced salt content of 25–50% without affecting critical quality attributes [84,88]. In other meat products, HPP at 150 MPa is also a viable technology for making low-salt breakfast to 1.5% in breakfast sausages without adverse changes in sensory quality [89]. Overall, the increase in saltiness in meat products is due to treatment-induced interactions between sodium ions and protein structures, resulting in a significant release of sodium on taste receptors on the tongue [87]. Furthermore, HPP can inactivate vegetative cells and bacterial spores in the complex food matrix [90]. This result is most likely due to low Water Activity (A_w_) due to high solute concentration, physical elimination of water via dehydration, or the presence of oil/fat. Therefore, HPP is a technology that doubles function in meat products by inactivating microorganisms and a technique to improve water binding, making HPP a promising technology in the food industry [83]. However, there are disadvantages to using this technology; its efficacy depends on the product’s characteristics and requires a high initial investment.

Moreover, immersion is a technology often used in the meat industry to increase the shelf life of products, flavor, juiciness, and softness compared to immersion in static techniques. At the time of immersion, the HPP approach can also increase the distribution of salt in meat more effectively, resulting in a stronger salt perception even when the real level of NaCl is low [91]. The rapid curing process also increases salt taste levels, providing advantages, such as better regulated enzymatic softening and lower levels of NaCl in immersion solutions, yet causing structural damage to soaked foods [92]. It is also similar to ultrasound intensity, which increases the time transfer of salt during immersion. The effects of ultrasound treatment on beef tissue also increase the NaCl gain rate perception [93,94,95].

##### High Hydrostatic Pressure (HHP)

High hydrostatic pressure (HHP) processing is also an effective nonthermal means of improving food safety and shelf life for meat products as a postprocess intervention [96]. For the HHP application in ready-to-eat fish products with a NaCl reduction of 25%, a feasible alternative is to employ UV-C at 0.310 J/cm^2^ or HHP at 300 MPa for 5 min, effectively maintaining the cooking loss, instrumental color, texture, and salty taste [97]. However, the interaction between changes in the conformational structure (secondary and tertiary structures) of meat product gel characteristics and product quality utilizing the HHP approach remains unknown [98].

##### Cold Processing Phases (CPP)

Another strategy was investigated by Pinna et al. [99], the strategy of cold processing phases (CPPs) in ham products. The CCP was made to produce ham with a 25% reduction in salt. The A_w_ decreases during the process, increasing the shelf life, while the color properties of the finished product are unaffected by the salt reduction and process modifications. Furthermore, proteolysis rises when the salt in the ham decreases, resulting in an increasingly softer texture. However, increased salt diffusion of the back skin may assist in compensating for the increased proteolysis of the bicep femoris muscle, which is depleted of salt during the decreased salt ham phase [99].

#### 3.1.5. Crossmodal Odor–Flavor Interaction Strategy

**Figure 4:**
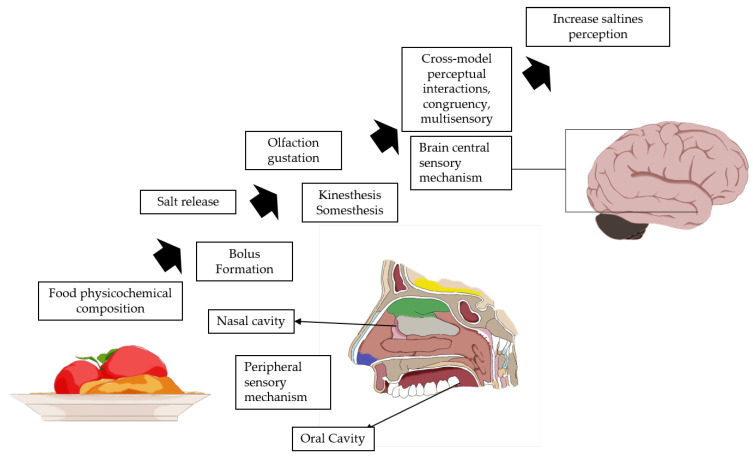
Experience crossmodal sequential processes to improve salty perception. Adapted from Ref. [4] with permission from the Royal Society of Chemistry.

Evidence of crossmodal integration between taste and odor is extensively provided. The enhancement of retronasal odors by a sweet stimulus is the result of an adaptive sensory mechanism designed to increase the salience of nutritive food flavors [100]. For instance, the aroma of strawberries enhances the sweetness of sweetened whipped cream [101]. Crossmodal odor–flavor interactions are also a way to enhance the saltiness of food through modifications caused by odors in taste perception. Thomas Danguin et al. [4] reported that salt-related odors could increase saltiness in a water solution with a low NaCl content. The increase in odor-induced salty perception (OISE) depends on the concentration of salt (intensity) (Figure 4). OISE is considered to be an efficient strategy to decrease salt content. However, its effect on texture depends on the low amount of salt in the solid version. Variance in nutrient matrix ingredients affects the release of salt and the general salty taste. Only models of foods with soft textures are found to increase saltiness significantly even though techniques that combine the heterogeneous stimulus and OISE are found in cream-based food systems to compensate for and reduce salt content by more than 35% without a substantial lack of acceptance [4].

### 3.2. Behavioral Strategy

#### 3.2.1. Memory Process Strategy

Memory processes influence eating behaviors, and efforts to improve memory of eating have produced varying degrees of success in reducing future eating [102]. Herbert et al. [103] analyzed the effects of various forms of repeated exposure to memory with low-salt broth flavor using memory processing techniques. The results showed that multiple experiences with test soups did not affect taste memory. However, the participants remembered that the final exposure soup was saltier than the low-salt preparations and recalled the salt concentrations associated with the individual’s ideal salt concentration [103]. This result could be a useful intervention to reduce overconsumption because it is related to improving eating memory [102]. However, little is known about factors that affect eating memory, especially salt intake.

#### 3.2.2. Gradual Salt Reduction Strategy

Gradual salt reduction investigated by Toft et al. [8] shows how the effect is tested using a linear mix model. Their study evaluated statistical differences among three fractions (gradually salt-reduced bread, salt-reduced bread, and dietary counseling to reduce salt intake further and increase potassium intake or standard bread). Other results showed that reduced salt consumption by lowering salt levels in bread with intervention alongside nutrients might improve salt flavor sensitivity, resulting in a preference for low-salt bread (0.4 g salt/100 g) [104]. In addition, the implementation of the salt reduction program has succeeded in gradually lowering salt levels in bread by 35% (from 1.7 ± 0.2 g/100 g to 1.1 ± 0.1 g/100 g) for three years without consumers noticing [105].

Moreover, one method that can be used in Salt Reduction Intervention (STRIVE) is to facilitate the evaluation of the gradual salt reduction strategy. Trial et al. [106] report that a STRIVE study was used to evaluate bread consumption on metabolic, chronic, and health impacts with decreased salt levels or accompanied by a nutritional counseling model. STRIVE is designed as an instructional tool for assessing and advocating adjustments in salt consumption. These findings reveal that the mechanism affects the sympathetic nervous system, the renin–angiotensin–aldosterone system, and the formation of salt preference limitations [106]. This method can be used to assess the gradual salt reduction strategy for various products to obtain more comprehensive results that are useful in the future.

#### 3.2.3. Swap to a Low-Salt Food Strategy

Swap, a strategy researched by Riches et al. [107], can reduce salt intake to give customers the option to switch to a low-salt diet during online shopping. They provide a broader range of salt-related alternatives to salt reduction rates. The salt reduction from the swap market is similar but with a minimum salt content for substantial salt reductions, including preferred foods. The first group received the same alternative with 5–20% less salt, while the second group received the same less salt swap and an option with >20% medium less salt. The results showed that providing replacement products with substantial salt reductions, such as theoretically different products, would not minimize acceptance and significantly reduce the salt content of the shopping cart [107].

Furthermore, He et al. [108] found that lowering salt intake led to a lower soft drink consumption. This result relates to the link between salt consumption and total fluid consumption. Salt is a key cause of thirst, and increasing salt intake will increase fluid consumption, mainly of sugary drinks [17,109]. Conversely, lowering salt intake may also reduce sugar intake, which is also good for health. However, further research into the relationship between salt reduction and sugar intake in other food categories is required because of the lack of data about salt’s direct effect on blood glucose levels [110].

## 4. Effects of Low Salt Content on Food Characteristics and Food Safety

### 4.1. Bread Products

Reducing the quantity of salt in food has a variable influence on food properties. For bread products, reducing salt levels (<1.2%) has an impact on decreasing dough resistance to extensibility and complex modulus without affecting the liquid–solid ratio [111]. In comparison, the significance of salt in a small amount (1.5%) in the reinforcement of the wheat gluten network (≤86%) increases dough gas retention and affects yeast activity [112]. However, from a taste perspective, the 10% reduction in NaCl in common brands of pizza dough is imperceptible [113]. The salt reduction directly impacts texture, which has implications for undesirable products. As a result, determining the precise decrease of salt content is critical for determining the rheology of bread. For instance, to prevent excessive expansion when the salt level is decreased, the dough base can contain starch with a high concentration of amylopectin [114].

Moreover, Diler et al. [81] found that a 25% salt decrease may be achieved by maintaining 50% of the salt in the dough to maintain the dough characteristics and retaining 25% as salt grains to produce a high saltiness area, hence raising the perception of the saltiness of the dough. It was accomplished by using the salt grain encapsulation technology to create very salty specks and optimizing the dust system to ensure a homogenous dispersion of the encapsulated salt grains in the dough during the laminating process [81]. This result aligns with the sensory contrast technique, which employs encapsulated salt crystals ranging from 1000 to 2000 μm, allowing for salt reductions of up to 50% while preserving customer preference for the bread product [80]. Furthermore, instead of simply depending on conventional salt reduction, it is expected that integrating different strategies will provide better products.

The salt concentration is also related to the formation of aroma in bread products. One of the sensory properties of the bread assessed its aroma, which describes several factors: the composition of the ingredients and yeast, the degree of mechanical and enzymatic damage caused by kneading and yeast, and the strength of thermal reactions that occur during baking [115]. Furans are usually caused by the oxidation of thermal sugars and the Maillard reaction, along with pyrrols, pyrazines, and strecker aldehydes, which are important to form the aroma of cakes or bread crusts [116]. The salt concentration significantly influences the volatile profile, which results in a higher methyl pyrazine 2-methyl furan concentration. Even though, if measured from the color aspect, strecker aldehydes and diacetyl (2,3-butanedione) in bread contain 20 g/kg of salt, Maillard browning is more critical at higher salt concentrations [115]

### 4.2. Cheese Products

In cheese products, salt levels and pH have the necessary effects on the rheological profile of cheese and the fat droplet scale [117]. The reduction of NaCl in cheese can reduce the cheese’s elasticity, while changing the cations from sodium to potassium can increase the cheese’s elasticity [23]. Moreover, salt affects complex ingredients or texture interactions in semisolid food, which influences how salty a food product is perceived [22]. Reducing salt concentrations also decreases insolubility significantly. Protein solubility decreases at a high ion strength, and the protein escapes from the solution [118]. With an increase in dry matter content, it was shown that the diffusion coefficient of NaCl (*D _NaCl_) reduced, which influences the growth of viscoelasticity and the reduction of cavity volume [119]. This result, aligned with increased dry matter content, resulted in a declining salt release, which reduced the perception of saltiness [117].

Furthermore, the proteins in cheese react with each other, fat, water, and salt, depending on the cheese’s manufacturing conditions and ionic atmosphere and the level of such interactions [120]. There is a link between the ionic strength of the salt type and protein solubility at different pH levels in protein-based foods [121]. A low protein content will affect the decrease in salt concentration in cheese related to the low solubility of casein in decreasing salt levels [122]. Additionally, low-protein cheese has a lower sodium-bound fraction and a longer relaxation time, which results in higher sodium mobility and fewer ionic interactions between casein and sodium molecules [123]. NaCl in cheese or protein suspension increases the ion potency of the system, solid behavior, shear-thinning, and frequency-dependent viscoelastic behavior [124]. In addition to its relation to protein, a low lipid/protein ratio makes cheese firm and hard, decreasing the sodium’s mobility during salt release [123]. This is consistent with the fact that adding fat to the protein gel system can increase saltiness by 26% [125].

In application, reducing salt by up to 50% boosted melting and slightly decreased stretch in mozzarella, whereas a 60% reduction in salt-restricted melting and consumer liking fell as salt was reduced [126]. In comparison to storage duration, the salt concentration has little effect on the texture properties of cheddar or the thawing and stretching of mozzarella cheese [126]. This result aligns with the salt content being less influential than pH on rheological behavior, dressage tribology, and sensory. Tribological behavior changes with time, and lower NaCl concentrations are becoming less acceptable to customers. While in another type of cheese, there are suggestions that reduced-salt cottage cheese sauce with 2.2% and 0.73% NaCl formulations at pH 5.0 is similar to the full-salt formulation [127].

Furthermore, salt content also significantly impacts A_w_ evolution and the microbiological profile survival in meat and cheese products related to food safety [5,6,7,24,128]. In cheese products, salt is essential because it maintains and controls lactic acid bacteria (LAB) growth of certain bacterial contaminants and pathogens in the final cheese. The water content is also related to most of the peptides identified and their salt concentrations. The salt reduction caused the ratio of peptides to proteinase activity to decrease significantly [129]. While in cheddar cheese, proteolysis and the overall speed of maturation are faster as salt concentrations decrease, and a higher percentage of salt decreases cause α_s_-casein degradation, yet no variation in the degradation of β-casein was identified [130].

### 4.3. Meat Products

Reduced salt in meat products has a different effect, especially on the structure, texture, and shelf-life of meat products. For meat products treated by ultrasound, the total liquid release is reduced along with the salt release. The sample with 0.75% salt displays microcracks in myofibrils and increased sensory acceptability of cooked ham [70]. This result aligns with removing sodium by 34.64% does not affect the properties of Bologna sausages, and the A_w_ values remained unchanged due to salt reduction, indicating that the salt substitute used did not affect the concentration of free water [131]. While a 1% reduction in salt reduced cooking loss, it increased moisture content, decreased fat levels, and produced a firmer, springier, and chewier final product than sausages with higher salt concentrations [132]. Salt also affects flavor, and palatability enhancers are employed to increase sensory features by attenuating bitterness and sweetness. A higher salt concentration (0.8–2.2%) in pork breakfast sausage has a higher level of customer acceptance than low salt content (1.4%) [133].

Pinna et al. [99] found increased proteolysis in reduced salt ham using the cold phase strategy, helping soften the texture. Furthermore, a combination of some additives can be used, such as microbial transglutaminase, as a preventive measure to prevent texture occurrence in meat and significantly prevent texture damage due to salt reduction [134]. Partial salt replacers, including L-his and L-lys, were also shown to lower Na by 53.79%, and another advantage is that lipid oxidation is delayed, resulting in an increased lipolysis and a higher free fatty acid concentration and higher phospholipase activity in the final stage of ripening dried loin [135].

In terms of meat product food safety and shelf life, salt usually employed in fermented meats prevents the growth of unwanted microorganisms while promoting the growth of salt-tolerant lactic acid bacteria [136,137,138]. Stringer and Pin [139] evaluated the implications of reducing salt in different foods based on pH, moisture content, and concentration of ham, bacon salt, smoked salmon, chicken rolls, cottage cheese, and beef burgers by modeling the growth of food pathogens (*Listeria monocytogenes, Yersinia enterocolitica,* and *Bacillus cereus*). The results revealed that the growth rate of foodborne pathogens was much higher in the reduced salt products than in the other products. Moreover, salt-sensitive organisms, such as *Clostridium botulinum*, did not grow in products containing 5.5% aqueous salt in this study, yet had the potential to grow in 4 weeks at 8 °C if the aqueous salt concentration is reduced to 2.85% [139]. This result is also related to the shelf life of meat, with lower NaCl content products having a shorter shelf life than those regularly formulated [39]. For example, low-salt bacon (2.3% *w*/*w* NaCl) has a shelf life of just 28 days, whereas control bacon (3.5% *w*/*w* NaCl) has a shelf life of up to 56 days [139].

The function of Na is critical to the product’s shelf life. Therefore, the salt replacer method is typically applied in this case. A reduction in NaCl of up to 40% in substituted cooked meat products with a commercial mixture of potassium lactate and sodium diacetate, for example, can extend shelf life for 6–7 days [140]. A similar result was also found in salami products. The replacement of NaCl with 1.6% potassium lactate (2.8% NaCl content) was successful in preventing microbiological growth without sacrificing product quality compared to salami products containing 4% NaCl [141].

The substitute component impacts the product’s taste, texture, and food safety, which depends not only on the type of replacer employed but also on the meat product and its formulation [142,143]. Therefore, proper consideration is needed in combining several strategies to maintain product quality. Table 1 summarizes the various salt reduction techniques and their impact on breads, meats, cheeses, snacks, fish, and seafood products.

## 5. Salt Reduction Effects on Consumer Acceptance

Salt used during food processing or preparation is the primary source of sodium. Salt influences not only the perception of saltiness but also the taste perception that determines food taste. Decreasing the salt content will reduce food acceptance related to food intake. The priority challenge is reducing salt concentration while maintaining consumer acceptability of food [25,26,117]. Therefore, it is essential to integrate all sensory information acquired throughout the application of the salt reduction strategy.

Each salt reduction strategy has a different impact on consumer acceptance. For instance, salt replacement using KCl has different effects on different food categories. Sensory properties are less preferred due to the bitterness and metallic taste of potassium salts. Partial salt replacement with 40% KCl in fermented sausage products results in flavor and texture defects while having no impact on microbiological stability [160]. In line with this result, replacement with >30% KCl has a significant flavor defect in dry-cured loin [159]. While salt replacement with KCl promotes syneresis in cheese products, only 25% have maximum sensory acceptance [128]. However, in bread products, partial salt replacement with 30% KCl has acceptable sensory characteristics [149].

Furthermore, in different processes, improvements in the consistency of reduced-salt bread with remilled salt did not affect its acceptance or consumer acceptance [115]. This result is in line with no significant instrumental variations and no visually observable color differences found for ham with salt replacement during preparation, nor was there any effect on customer acceptance [70]. Moreover, ultrasound treatment impacts the improved sensory acceptance of cooked ham altered with 0.75% NaCl [70]. Replacing 60% NaCl with flavor enhancers affects emulsion stability, microstructure, and consumer acceptance of Bologna sausage [131]. This result aligns with the fact that NaCl removal affects the microstructure of Bologna sausages and the effect on consumer acceptance, along with the consistency of emulsions and instrumental textures [70]. Meanwhile, in cheese products, cheeses with a 50% reduction in salt have less sensory acceptance and are less stable, while cheeses with a 25% reduction in salt resulted in a similar firmness, peptide profile, and sensory approval relative to regular cheeses [151]. Overall, some researchers report decreasing salt content in various food products and processes with different effects on consumer acceptance. In conclusion, more specific research is needed for each product and process with a reduced salt strategy to meet the product criteria consumers expect.

## 6. Conclusions

Changes in food production practices have minimized possible health risks, but diverse factors affect how customers perceive salt in food. Reducing salt content will benefit food companies by increasing food quality without affecting customer acceptance and meeting prescribed daily intake limits. Prominent food industry strategies have included technological strategies (salt replacement, food reformulation, size and structural changes, and alternative processing). These varied strategies have also been widely implemented, particularly on products with high salt contents, such as bread, cheese, meat, soup, fish, and seafood. This result is demonstrated by the numerous findings from various research studies that have been published.

The application of diverse strategies affects each product category differently due to changes in product qualities, such as solid, semisolid, and liquid. Because of this discrepancy, choosing the best technique for decreasing NaCl in the food is challenging. As a result, it is critical to understand the fundamental principles of product processing, the interaction of the components that comprise the product, and the factors that influence consumer taste perceptions. Thereby, the decision of the suitable strategy and a deeper understanding of its effects on the various physical properties of salt will give salt tremendous potential to be structurally altered and ultimately involved in the production of salt-reduced food products.

## Figures and Tables

**Table 1:** Summary of various treatments to reduce salt levels and their impact on food characteristics.

Food Category	Strategy	Treatment	Characteristics of Food Effects	Reference
**Bread Products**				
Durum wheat bread	Reduced Salt	Decrease of 50% NaCl (10–20 g/kg NaCl).	Less intensely colored crust and a weaker toasted aroma positively affected bread-specific volume and crumb consistency.	[115]
Wheat bread	Partial salt substitute	Addition of 1.5–3% salt substitute Pansalt^®^ (NaCl 57%, KCl 28%, MgSO4 12%, lysine hydrochloride 2%, silica 1%, and iodine 0.0036%).	Similar effects on bread control sensory attributes, yet unable to maintain the same level of perceived saltiness, produced a perceptible increase in bitter taste and aftertaste in the crust.	[144]
Brown bread	Partial salt substitute	Salt substitute with potassium (K) (55.2%), magnesium (Mg) (69.0%), and (Ca) calcium (34.8%).	Baking quality, appearance, texture, and taste are acceptable and achieved 32.3% reduced sodium.	[45]
Wheat bread	Partial salt substitute	Substitution of 40% salt with potassium (K) or calcium chloride (CaCl_2_) or magnesium (Mg) salts.	There is no negative impact on the rheology of the dough.	[145]
Wheat flour	Partial salt substitute	Addition of 25% substitution of KCL, MgCl_2,_ and CaCl_2._	No difference in the dough production timing and the dough’s stability increases.	[146]
Bread	Partial salt substitute	Replacement: -75% by Na-gluconate -50% by K-gluconate -100% by Na-gluconate/K-gluconate	-In partial replacement (75% and 50% Na or K gluconate), there was no change in the bread rheology or volume, nor was there any significant effect on overall desire. -In 100% replacement, decreased resistance to extension	[147]
Ground beef patties	Salt mixture	Addition of low-sodium salts with 2% Pansalt^®^ (PS)	Detrimental impact on sensory quality of ground beef bread made using Pansalt^®^ combination compared to bread containing NaCl.	[148]
Bread	Partial salt substitute	0.3% addition of KCl combined with glutamate	Because it covers the bitter aftertaste, it is acceptably sensory and achieves 75% reduced NaCl	[49]
Bread	Partial salt substitute	Potassium (K) salt replaces 30% sodium	Sensory characteristics are acceptable	[149]
Wheat bread crust	Coarse-grained NaCl	In addition, coarse-grained NaCl (2−3.5 mm)	Increased saltiness as a result of sensory contrast, yet faster sodium release during mastication while preserving taste quality and achieving 25% reduced NaCl	[79]
Pizza crust	Partial salt replacement	Replace 30% NaCl by KCl or coarse-grained NaCl (0.4–1.4 mm)	Enhancement of saltiness through taste contrast and an accelerated sodium delivery measured and achieved 25% reduced NaCl.	[150]
Bread	Encapsulated salts	Encapsulated salt crystals 1000–2000 μm	No apparent loss of the salty flavor and achieved 50% reduced NaCl	[80]
Sheeted dough	Encapsulated salts	Holding 50% of the salt in the dough recipe to maintain the dough properties and save 25% as salt grains	Enhance the saltiness perception and achieve 25% reduced NaCl	[81]
**Cheese Products**				
Prato	Salt reduction	25% and 50% salt reduction	-25% reduction has a similar peptide profile, hardness, and sensory acceptability. -50% salt reduction was less firm and less sensory acceptable than the control cheese	[151]
Mozzarella	Salt reduction	50–60% salt reduction	Lowering salt by up to 50% boosted melting and slightly reduced stretch, whereas reducing salt by 60% inhibited melting.	[126]
Cheddar	Salt reduction	Salt reduction of 0.5–3%	Reducing salt has a negative impact on the taste and texture.	[152]
Cheddar	Partial mineral salt replacement	Addition of 298–388 mg CaCl_2_ and MgCl_2,_	Significant off-flavor in cheese (bitter, soapy, and metallic taste)	[153]
Mozzarella	Partial mineral salt replacement	Addition of <25% KCl	It has a higher pH, metallic taste, and moisture content than cheeses with a higher K concentration. However, it melted with less hardness.	[154]
Cheddar	Partial replacement of mineral salts	Addition of 60% low-sodium mixture of NaCl and KCl	Adding KCl at a level that maintains A_w_ leads to a slight bitterness, controllable salinity, and acceptable consumer acceptance. The same effective salt-to-moisture ratio.	[155]
Cheese	Partial salt replacement-based emulsifying salts	Application of hydrocolloids: -Modified starch (with bound sodium octenyl succinate) -Low methoxyl pectin (alone or combined with lecithin) -Locust bean gum, k-carrageenan, and i-carrageenan	The products containing 1% (*w*/*w*) ic-carrageenan or i-carrageenan were homogenous but hard with a fracturable texture.	[156]
White cheese	Partial salt replacement	Application of hydrocolloids: guar gum, carrageenan, xanthan gum, and gelatin	Reduced salt in the brine ≤8% caused no defects because stabilizers prevented water entry into the cheese by retaining water.	[157]
Feta cheese	Salt replacement and alternate processes	Addition of KCl and milk ultrafiltration treatment at a volumetric concentration factor of 4.5:1	Adding KCl promoted syneresis, and only 25% replacement by KCl had the maximum sensory acceptance.	[128]
Processed cheese	Partial salt replacement	Xylooligosaccharide (XOS), salt reduction, and taste enhancers (arginine and yeast extract) addition.	Enhanced the rheological, physicochemical, and sensory attributes.	[158]
**Meat Products**				
Dry-cured loin and fermented sausage	Partial salt replacement	KCl, potassium lactate, and glycine addition	Significant flavor defects were detected with replacement of >30% in both products replaced with K-lactate and KCl, and loss of cohesiveness at a replacement rate of >50% with glycine and > 30% with K-lactate.	[159]
Fermented sau-sage	Partial salt replacement	Addition of KCl (40%), K-lactate (30%), and glycine (20%) addition	Resulting in flavor and texture defects and having little effect on microbiological stability	[160]
Packaged cooked meat	Partial salt replacement	Sodium diacetate, potassium lactate, and combination 2–3% addition.	Sensory quality and shelf life were increased while lowering NaCl levels by 40%.	[140]
Ham	Partial salt replacement	70:30% NaCl:KCl or 70:30% NaCl:MgCl_2_	No organoleptic or quality changes were observed compared to control.	[161]
Fermented cooked sausages	Partial salt replacement	KCl concentrations of 50% and 75% substitute and MSG, disodium guanylate, lysine, disodium inosinate, and taurine are added	Masking unpleasant flavors produced by lower salt levels	[162]
Chicken breast meat	Partial salt replacement	Sodium tripolyphosphate and β-glucan addition and HPP at 40 °C and 600 MPa pressure	There is a negligible effect on color properties.	[163,164]
Bologna sausage	Partial salt replacement	Citrus fiber addition	Most physical, chemical, and sensory aspects did not change.	[165]
Ready-to-eat chicken breast	Partial salt replacement and alternative processing	Replacing 50% NaCl with KCl and HHP at 600 MPa for 3 min	The salt replacement did not affect the microbial counts, and HHP processing improved the hardness and sensory attributes of the sodium-reduced	[84]
Dry cured loin	Partial salt replacement	The salt substitute contained 39.7 g/100 g of NaCl, 51.3 g/100 g of KCl, and a mixture of L-histidine and L-lysine (9.0 g/100 g)	Decrease of 53.79% in Na content delayed lipid oxidation and produced slightly higher lipolysis, resulting in larger content of free fatty acids and higher phospholipase activity	[135]
Chicken meat batters	Alternative processing strategy	Heat under pressure (HUP) treatment at 200 MPa 75 °C, 30 min	Improved the gel qualities, resulting in glossy coarse, loose gels with high water loss, and low acceptability.	[86]
**Snack Products**				
Shoestring potatoes	Reducing the size of particle salt mixture	Reducing particle sizes of salt mixture (NaCl, MSG, and KCL) of 60 μm and 88 μm	No, significantly changing the sensory quality and achieved a sodium decrease of 69%	[166]
Shoestring potatoes	Reducing the size of particle salt	Reducing particle sizes of 26 µm particles	Maintained the same perception of salty taste and sensory quality and achieved a sodium decrease of 51%	[167]
Cheese crackers	Reducing the size of particle salt	Reducing 3 logs from regular salt to nano spray-dried salt	Maintained low counts of yeasts and absence of molds, did not adversely influence sensory quality attributes and achieved a sodium decrease by 25–50%	[168]
**Soup Product**				
Tomato soup	Salt reduction	Internalized salt solution stabilized with nonchemically modified waxy rice starch (WRS) and octenyl succinic anhydride (OSA)	Enhanced for gelatinized WRS compared to OSA starch stabilized emulsions and achieved a sodium decrease of 25%	[61]
**Fish and Seafood**				
Cooked fish batter	Salt reduction and alternative processing	The isolated and combined effect of UV-C (0.310 J/cm^2^) and high hydrostatic pressure (HHP; 300 MPa for 5 min at 25 °C)	The treatments did not affect sodium chloride concentration, redness, yellowness, cohesiveness, springiness, or resilience and were reduced by 25% NaCl.	[97]
Smoke-Flavored trout	Salt mixture and alternative processing strategy	Substitution of NaCl with 30% combined with spray dryer	Higher hygroscopicity and saltiness because of their lower bulk density and existence of agglomeration, surface roughness, and macro pores.	[64]
Smoke-flavored salmon	Partial salt replacement	Addition of 50% KCl with smoke flavoring by water vapor permeability bags	It did not significantly affect the quality and shelf-life	[169]
Cold-smoked salmon	Salt reduction and alternative processing	Sodium-reduced samples (2.7–3.7 g salt/100 g) with cold smoking + vacuum packaging	Regarding aerobic and anaerobic mesophilic counts, organoleptic properties, texture, color, and the growth of *Listeria monocytogenes* did not differ significantly from the commercial reference product	[170]
Fish ball	Partial salt replacement	Addition of 20% KCl +15% sucrose +15% citric acid with 25% + corn flour + 75% peanut flour or 25% barley flour +75% pea flour	Physicochemical and sensory evaluation, emulsion stability, cooking yield, and overall acceptability	[171]
Fermented fish	Partial salt replacement	Addition of 25% and 50% KCl	Higher hardness, adhesiveness, and springiness	[172]
Salmon	Partial salt replacement	-70% NaCl + 30% KCl -50% NaCl + 50% KCl -70% NaCl + 20% KCl + 10% CaCl_2_ -70% NaCl + 30% KCl + 5% yeast -70% NaCl + 30% KCl + 0.25% taurine	-70% NaCl with KCl had lower sensory damage -50% KCl depicted the best a* value -Treatment with the addition of CaCl_2_ exhibited the highest L* value, highest springiness, hardness, and chewiness -Addition of yeast extract best improved the sensory defects caused by KCl -Addition of flavor enhancers could improve the poor flavor of the salted salmon caused by the KCl addition	[52]
Fermented shrimp paste	Partial salt replacement	In addition, 25 and 50% KCl	Reduced lipid oxidation, oxidative rancidity, and antioxidant activities were maintained.	[173]
Sushi (tuna and shrimp)	Salt microspheres	-Addition of 87–99% hollowed microsphere of regular salt crystals -Addition 38% KCl	-The quality of sushi products made from tuna or shrimp was preserved by hollowed microsphere salt -KCl addition improved the bitterness in maki shrimp and reduced the saltiness in nigiri tuna.	[76]
Seabass sausages	Salt reduction and partial salt replacement	-50% NaCl + 50% oleoresins microcapsules -50% NaCl + 50% KCl	-Replacement of 50% NaCl with KCl microcapsules or oleoresin showed the best results in reducing Na content (30.9–36.3%) while maintaining sausage quality. -Substitution with KCl resulted in a product richer in K (997.2 mg/100 g)	[174]
Smoked sea bass (*Dicentrarchus labrax L.)*	Partial salt replacement and cold smoking	Addition of 50% NaCl + 50% KCl	Effective in preventing lipid peroxides and keeping the total volatile basic nitrogen value is below the decay threshold. Salt substitution with the K did not change the quality of smoked fillets.	[175]
Smoked salmon (*Salmo salar)*	Partial salt replacement	-75% NaCl + 25% KCl + 0.1% commercial masking agent -50% NaCl + 50% KCl l+ 0.1% commercial masking agent	No significant difference in physicochemical properties in the smoke sample with 50% KCl, while the sample with 25% substitute did not show a difference with control (100% NaCl)	[170]
Salmon pate (*Salmo salar*)	Partial salt replacement	20% NaCl + 80% substitute with KCl	Substitution of 80% with Saltwell^®^ at a reduction of 22% sodium does not affect microbial activity. In comparison, there were small differences in three of the twelve sensory attributes evaluated (coherent texture, salty taste, and canned fish taste).	[176]

## Data Availability

Not applicable.
